# The Benefits and Challenges of Implementing Teleophthalmology in Low-Resource Settings: A Systematic Review

**DOI:** 10.7759/cureus.70565

**Published:** 2024-09-30

**Authors:** Imran Ahmed Khan, Md. Abu Bashar, Alka Tripathi, Neha Priyanka

**Affiliations:** 1 Community Medicine, Baba Raghav Das Medical College, Gorakhpur, IND; 2 Community and Family Medicine, All India Institute of Medical Sciences, Gorakhpur, IND; 3 Ophthalmology, All India Institute of Medical Sciences, Gorakhpur, IND

**Keywords:** cost-benefit analysis, health expenditures, ophthalmology, resource-limited settings, telemedicine

## Abstract

Technology has significantly changed medical practice, including diagnosis, treatment, and availability. Telemedicine use in the specialty of ophthalmology seems to be a promising field. In underserved populations, limited coverage of ophthalmic healthcare facilities results in a higher burden of eye-related diseases and visual impairment. The main obstacle preventing these individuals from receiving eye care consultations is difficulty in access and transportation. There is an urgent need for eye care facilities for these people, and teleophthalmology has the potential to provide eye care facilities to these underserved people. Teleophthalmology was reported as cost-effective, time-saving, reliable, and efficient for underserved populations. However, teleophthalmology has certain limitations in its implementation in the form of a high initial cost of equipment, problems with consistent electricity and internet supply, and the reluctance of people in certain regions toward acceptance of teleophthalmology. This systematic review assessed the benefits and challenges of implementing teleophthalmology in low-resource settings.

## Introduction and background

Telemedicine is defined as providing healthcare services through electronic communication and information technologies where healthcare providers and recipients are at different geographical locations [1]. Telemedicine has the potential to significantly improve the health sector by allowing real-time communication and connecting patients with healthcare providers, storing and transferring health data, and remote patient monitoring and follow-up [2]. Telemedicine is also expected to reduce the burden on hospitals, the suffering of patients, the need for transport, hospital fear, and long queues. It saves out-of-pocket expenditure and time and increases the quality of health care with high satisfaction [3].

Teleophthalmology, the use of telecommunication technology in the remote diagnosis and management of almost all eye conditions to provide ophthalmic care, has shown promise in improving access to eye care services, particularly in underserved and low-resource settings [4]. Teleophthalmology helps in saving time and money involved in travel to seek the opinion of an ophthalmologist [5-7]. Teleophthalmology was found to be a to be a very useful tool during COVID-19 [8]. Moreover, COVID-19 has hastened the development and embracement of teleophthalmology [9].

However, its implementation in low-resource settings faces several challenges [10,11]. This systematic review aims to explore the benefits and challenges faced in implementing and scaling up teleophthalmology in such contexts. The primary objective of this review is to identify and analyze the benefits and challenges associated with the implementation of teleophthalmology in low-resource settings. The findings of this review could help policymakers and healthcare providers to have an evidence-based overview of different bottlenecks in teleophthalmology implementation and to formulate appropriate action plans.

## Review

Search strategy

The PRISMA (Preferred Reporting Items for Systematic Reviews and Meta-Analyses) statement for reporting systematic reviews was used for data reporting in this systematic review [12]. A comprehensive search was conducted using PubMed, Medline, CINHAL databases, and Google Scholar. Searches of the electronic databases were performed during July 2024. The search terms included "Teleophthalmology," "telemedicine," "eye care," "low-resource settings," "challenges," and "solutions." The search was limited to articles published in English from January 2015 to June 2024. The following search term combination was used in PubMed: ("Teleophthalmology" OR "telemedicine in ophthalmology" OR "remote eye care" OR “Tele-Eye Care” OR “Telehealth in ophthalmology” AND ("low-resource settings" OR "developing countries" OR "rural areas” OR “Resource-limited settings” OR “Underserved areas”). Data screening was used initially through keywords, titles, and abstracts. Finally, the relevant articles were selected for inclusion by reviewing the full texts of the eligible studies. An advanced search of Google Scholar was used to obtain relevant articles. Further, the reference lists of the studies whose full-text retrieval was done were also screened (citation tracking) to find additional relevant studies.

Selection criteria

Inclusion criteria were studies focusing on teleophthalmology implementation conducted in low-resource settings addressing benefits and/or challenges. Only peer-reviewed original articles and case studies were included. Articles not available in English, conference abstracts, editorials, dissertations, opinion pieces, and review articles were excluded. The keywords, title, and abstracts were assessed independently by two (IAK, Imran Ahmed Khan and MAB, MD Abu Bashar) reviewers. Discrepancies regarding the inclusion of studies were resolved after discussion and consultation with the third author, AT. The same reviewers also performed the full-text screening independently to decide which articles fulfilled the inclusion criteria. The senior authors MAB and NK (Neha Priyanka) resolved discrepancies regarding the inclusion of studies after a thorough discussion.

Data extraction and synthesis

Data were extracted using a standardized format, including authors, year of publication, type of study, focus of study, and conclusions. The benefits and challenges pointed out in the included studies were compiled in a table to find the common characteristics. Thematic synthesis was employed to discuss challenges in the implantation of teleophthalmology.

Results

**Figure 1:**
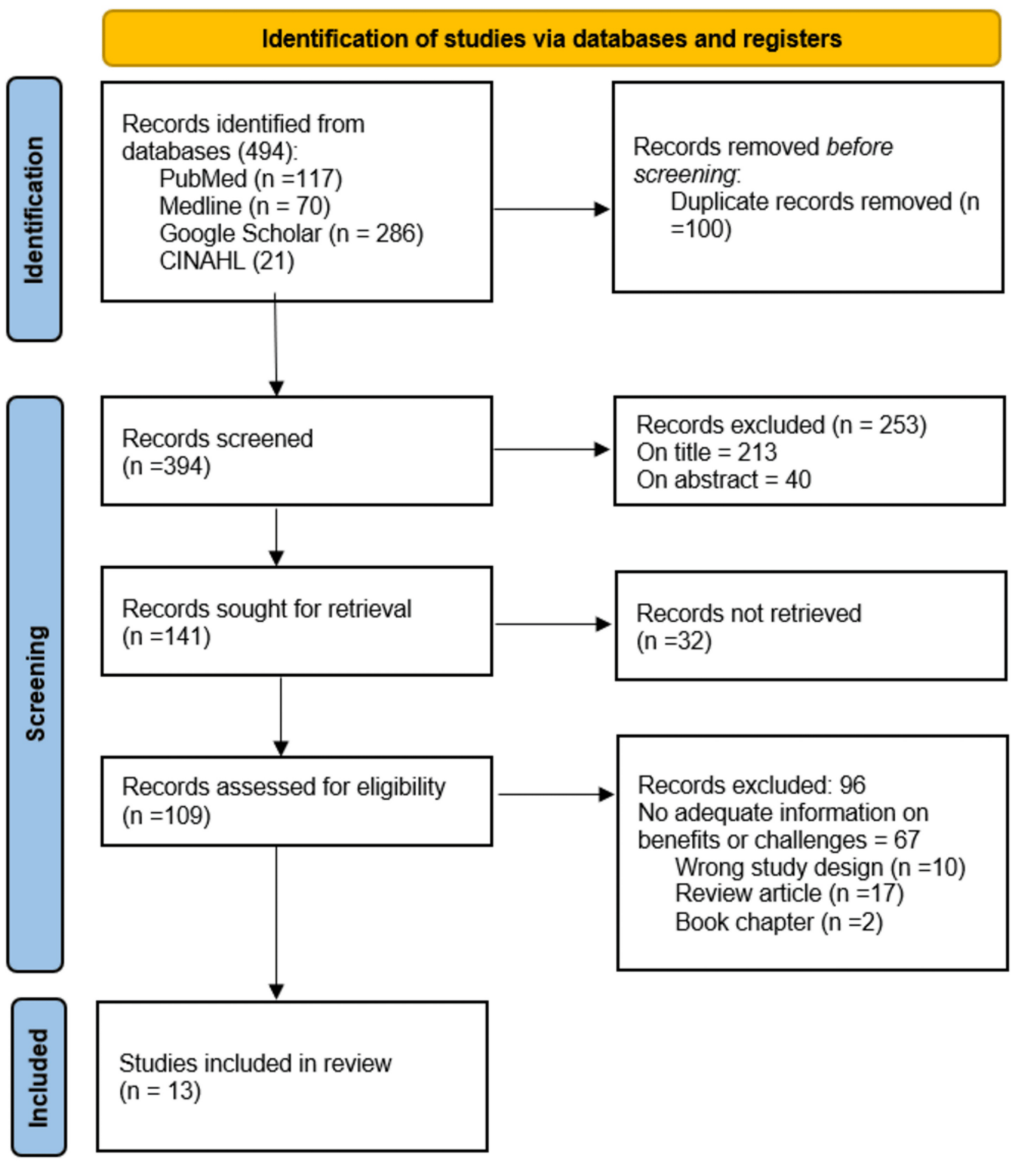
Preferred Reporting Items for Systematic Reviews and Meta‑Analyses (PRISMA) flow chart of search strategy

A total of 494 potentially relevant articles were identified from PubMed (117), Medline (70), CINAHL (21), and Google Scholar (286). After removing 100 duplicate articles, 394 were available for title and abstract screening. The screening through titles and abstracts further removed 253 articles. Out of the remaining 141, the full text of 32 articles was not retrieved and was removed. The full text of the remaining 109 articles was retrieved and assessed, and based on the selection criteria, 96 articles were excluded at this stage. Finally, the remaining 13 articles were selected for the systematic review presented in Figure 1.

The summary of the included articles is presented in Table 1.

**Table 1:** Summary of included articles CSME: clinically significant macular edema; EMR: electronic medical record; OTs: ophthalmic technicians; AI: artificial Intelligence; DR: diabetic retinopathy

Sr No.	Author (year)	Country	Article type	Focus area	Conclusions
1	Blackwell et al. [13]	Australia	Communications in Medicine	Acute ophthalmological problem requiring a specialist opinion	Teleophthalmology is well suited for the diagnosis and management of acute conditions and postoperative assessment of patients in remote areas.
2	Peter et al. [14]	Switzerland	A pilot study	Screening for DR using telemedicine	The sensitivity of detection of CSME by photography was considerably better than for live link telemedicine.
3	Bai et al. [15]	India	Original article	All eye conditions	Teleophthalmology was gaining more acceptance and other states also followed its implementation.
4	Pérez et al. [16]	South Africa	Project evaluation	Ocular opportunistic infections among the HIV-infected population	Despite technological barriers, teleophthalmology was found to be a useful approach to building clinicians’ capacity for fundoscopic examination, ensuring quality and continuity of HIV care, and preventing permanent vision loss and other morbidity among HIV-infected people.
5	Kim and Driver [17]	Canada	A mixed methods evaluation monitoring	The project focused on retinal health and eye disease	Teleophthalmology was a welcome addition to health services by the First Nations communities.
6	Thomas et al. [18]	Canada	A cost-effectiveness analysis was	Costs and benefits analysis of teleglaucoma as a screening device for glaucoma	Teleglaucoma resulted in improved health outcomes, increases access to ophthalmic care, and improves ocular healthcare service efficiency in rural areas.
7	Das et al. [19]	India	A retrospective study	Utilization of the eyeSmart EMR app	The eyeSmart EMR app used in teleophthalmology helps in connecting patients in rural areas and appropriate management of the patient by ophthalmologists.
8	Collon et al. [20]	Nepal	Original clinical study	Detection of ocular pathology and medical decision-making by an ophthalmologist of images captured by the mobile device-based ophthalmic camera by OTs in village screening camps	Teleophthalmology utilizing a mobile device ophthalmic camera system can provide high-quality images in the eye screening camps helping in detecting ocular pathologies.
9	Charlot et al. [21]	France	Retrospective, cross-sectional	Mobile screening for DR	The screening technique was found meaningful to detect earlier curable diseases in a population devoid of sufficient ophthalmologic care.
10	Chia and Turner [22]	Australia	Perspective	Based on our rural telemedicine experience identified key themes for successful implementation	Advances in AI have shown promise toward bridging the gap between expanding eye care demands and limited resources. This may be applied to urban centers to deal with surgical wait lists.
11	Patil et al. [23]	India	Retrospective analysis and a prospective telephone-based study in DR patients	Patient follow-up in a teleretinal screening program and finding potential barriers to care	Providing health education and improving referral strategies are essential to improve outcomes.
12	Barbieri et al. [24]	Brazil	Retrospective study	This study assessed different screening approaches in the Brazilian public healthcare setting	The telemedicine approach significantly reduced the waiting time for specialist evaluation in a real-world setting.
13	Li et al. [25]	United States	Qualitative study	Implementation process barriers and facilitators to using asynchronous telespecialty care	Feedback from the primary care team improves telespecialty service delivery for rural veterans.

Table 2 shows the reported benefits and challenges from the included articles. Teleophthalmology offers a precious, cost-effective solution for the diagnosis and timely treatment of most eye-related diseases. Enabling real-time consultations and identifying ophthalmic pathologies reduces the need for long-distance patient transport, minimizes healthcare expenditure, and prevents loss of daily wages. This technology effectively captures at-risk patients who might never receive an ophthalmic evaluation, ensuring prompt care without unnecessary delays.

**Table 2:** Benefits and challenges of teleophthalmology implementation

Benefits	Challenges
Allow definitive diagnosis of almost all eye-related diseases	Challenges of connectivity and power in rural areas
Significant reduction in waiting times for specialist evaluations	High initial capital expenditure
Facilitates initiation of treatment without any undue delay	Organizational barriers
Reduced expenditure and loss of daily wedges	Technological barriers
Cost-effective, particularly in rural areas, limiting the transport of patients over long distances	Poor communication between telehealth providers and the patient after tele-screening
Real-time consultation is feasible	Scarcity of properly trained staff
Identify and triage anterior segment pathology	Little awareness among the community
Good patient and healthcare worker satisfaction	Lack of awareness about value of regular follow-up examinations
Tele-retinal screening is a highly valuable technology that captures at-risk patients who might have otherwise never received an ophthalmic evaluation	Limited role in the detection of and referral for posterior segment pathology

Teleophthalmology faces significant challenges, including road and internet connectivity, power supply issues, high initial capital expenditure, and limited community awareness about the technology. The scarcity of properly trained staff and organizational and technological barriers further complicate its implementation. Additionally, the technology is limited in detecting and referring posterior segment pathology, and there is often an incomplete understanding of disease management. Poor communication between patients and providers after telescreening, combined with a lack of awareness about the importance of regular follow-up exams, also poses a barrier to effective care.

Discussion

Teleophthalmology ensures access to eye care by leveraging technology, particularly for remote and rural populations where ophthalmic consultation facilities are scarce or unavailable. It has shown improving access to eye care, including but not limited to conditions like retinopathy of prematurity, diabetic retinopathy (DR), glaucoma, and age-related macular degeneration. However, its adoption is faced with several challenges. Through this systematic review, we intended to determine the benefits, challenges, and possible solutions for implementing teleophthalmology in low-resource settings.

Benefits

Teleophthalmology allows definitive diagnosis of almost all eye-related diseases [15]. Ocular opportunistic infections among the HIV-infected population can be easily tracked and managed using teleophthalmology [16,26,27]. Teleophthalmology is cost-effective, particularly in rural areas, limiting the transport of patients over long distances and providing real-time consultation [18,21,26,28]. Screening of different eye conditions and triage with suitable referrals can be accomplished with the help of teleophthalmology [20,23]. DR and other eye conditions can be effectively screened by trained paramedical optometrists using simple modifications in smartphones with good inbuilt cameras and a few mobile applications [29-32]. A few studies found that telemedicine is suitable for treating patients with eye problems with good satisfaction of both patients and staff [13,17,33]. Teleophthalmology using AI has shown a good impact on expanding eye care demands, particularly in limited resource settings and reducing wait lists [22,24].

Challenges

The initial and ongoing operation of teleophthalmology face significant challenges. In a pilot study, the sensitivity of detection of clinically significant macular edema by photography was considerably better than for Livelink telemedicine, showing the superiority of examination of the patient by an experienced ophthalmologist [14].

Financial challenges

High initial setup and maintenance costs, coupled with limited funding and financial incentives, are some important financial barriers [15]. The device-dependent nature of teleophthalmology and the need for robust information and communication technology (ICT) infrastructure are significant barriers, especially in low- and middle-income countries (LMICs) [16,19,31]. Insurance companies are less likely to reimburse teleophthalmology services, which affects program sustainability [34,35].

Workforce related challenges

There is a shortage of trained ophthalmologists and support staff. Image analysis relies heavily on expert interpretation, which can be a bottleneck in resource-limited settings [16,25]. Inadequate training in teleophthalmology techniques is another important issue [36].

Technological challenges

Limited internet connectivity and bandwidth, lack of access to advanced imaging and diagnostic equipment, and inconsistent electrical power supply are noticeable considerations for the proper deliberation of telemedicine use in eye care [16,24,31].

Social challenges

Resistance to adopting new technologies and low patient awareness and acceptance of teleophthalmology are additional issues in rural areas [15]. Both patients and primary care providers (PCPs) often lack familiarity with teleophthalmology, leading to misconceptions and underutilization. Patients may have logistical challenges and misconceptions about diabetic eye screening, while PCPs struggle to identify when patients are due for screening [23,26].

Table 3 presents a strengths, weaknesses, opportunities, threats (SWOT) analysis of implementing teleophthalmology in low-resource settings, which should help in understanding the potential impact and challenges of this venture.

**Table 3:** SWOT analysis of teleophthalmology implementation in low-resource settings SWOT: strengths, weaknesses, opportunities, threats

Strengths	Weaknesses
Accessibility to remote and underserved areas	Technological limitations
Continuity of care for vulnerable populations	Patient acceptance and cultural resistance
Cost-effectiveness by reducing travel and in-person visits	Lack of training and expertise among healthcare providers
Efficient use of specialist resources	Data security and privacy concerns
Opportunities	Threats
Innovation in low-cost, portable devices	Questionable sustainability without ongoing funding
Public-private partnerships	Regulatory hurdles in telemedicine
Alignment with global health initiatives	Barriers to new technology adoption
Educational outreach to train local healthcare workers	Infrastructure challenges (e.g., poor electricity supply, unstable internet connection)

Potential solutions to challenges

Various potential steps may help overcome the challenges associated with teleophthalmology, making it a more effective and accessible tool for remote eye care. Advances in imaging technology and the use of portable devices make it easier to capture high-quality images, which can be analyzed remotely by experts [37]. The integration of artificial intelligence and image processing software can enhance diagnostic accuracy and efficiency, making teleophthalmology more effective [22]. Ensuring financial viability through clear patient coverage and care provider reimbursement policies is necessary for the sustainability of teleophthalmology programs. The establishment of public-private partnerships to share costs and resources is another domain of action [38]. Effective coordination of care between primary care and eye care providers is essential to improve follow-ups and manage referrals efficiently. Improved patient and provider education about teleophthalmology can increase its use. This includes addressing misconceptions and familiarizing both groups with the technology and its benefits [39].

The limitations of this systematic review include the limited availability of high-quality studies from low-resource settings. The inclusion of only English-language articles may lead to potential language and publication bias. Another important limitation is the heterogeneity in study design and outcomes, which complicates synthesis. Additionally, rapid technological advancements in the field may render older studies less relevant, and excluding gray literature could omit some important information.

## Conclusions

Teleophthalmology holds significant potential for improving eye care access in low-resource settings. However, its successful implementation on a larger scale requires a holistic approach addressing technological, financial, workforce, and social challenges. Implementing teleophthalmology on a larger scale in low-resource settings is a complex but achievable goal. Addressing technological challenges requires investment in infrastructure and the development of low-cost, portable diagnostic tools. Innovative funding models and cost-sharing partnerships can mitigate financial challenges. Workforce challenges necessitate comprehensive training programs and incentives for adopting teleophthalmology practices. Targeted education and engagement strategies can overcome social challenges.
